# A primary Rosai-Dorfman-Destombes disease of the scalp: case report and literature review

**DOI:** 10.3389/fneur.2023.1172695

**Published:** 2023-06-08

**Authors:** Wenxiong Song, Feiyu Ding, Yong Xiao, Xinhua Hu, Kun Yang, Liangyuan Geng, Yuanjie Zou

**Affiliations:** Department of Neurosurgery, Affiliated Nanjing Brain Hospital, Nanjing Medical University, Nanjing, China

**Keywords:** Rosai-Dorfman-Destombes disease, scalp, cutaneous, surgery, benign histiocytic proliferative

## Abstract

**Background:**

Rosai-Dorfman-Destombes disease (RDD) was first described in 1965 as a benign histiocytic proliferative disorder of unknown cause. Cases of RDD limited to cutaneous tissue have been reported over the past few decades, but single cutaneous RDD of the scalp is rare.

**Case presentation:**

We report a 31-year-old male with a lump on the parietal scalp without extranodal lesion lasting 1 month with gradual enlargement. The surgical incision ruptured with purulent after the first resection. Then the patient was treated with plastic surgery after disinfection and antibiotic treatment. Finally, he recovered well and discharged after 20 days.

**Conclusions:**

RDD of the scalp is rare. Surgical incision can cure the lesion but it may become infected because of increased lymphocytic infiltration. Early diagnosis and differential diagnosis of RDD are necessary. For treatment, individualized therapy is critical to patient prognosis.

## Introduction

Rosai-Dorfman-Destombes disease (RDD) is a non-neoplastic, benign, idiopathic non-Langerhans cell histiocytosis presenting in children and young adults. It mostly affects cervical lymph nodes, but approximately 40% of cases have extranodal lesions (1). Destombes first reported this disease in 1965 (2), then Juan Rosai and Ronald Dorfman described this disorder 4 years later (3, 4). RDD is considered primary cutaneous RDD (CRDD) if it is localized to the skin or subcutaneous tissue, and there are no systemic symptoms (fever, night sweats, weight loss). Because of its unique epidemiological and clinical features, it has been placed in the ‘R group' in the classification of histiocyte disorders (5).

There are few reported cases of RDD of the scalp, so we here report a case with RDD of the scalp the lesion was resected and xanthogranuloma was found in it.

## Case presentation

A 32-year-old man was found a lump on his parietal scalp. He sought medical treatment after 1 month, during which the lump gradually grew larger. He claimed the mass produced no pain. Clinical examination found that the mass was hard, smooth, and not easily moved under the skin. It measured 6 cm × 5 cm in area.

**Figure 1:**
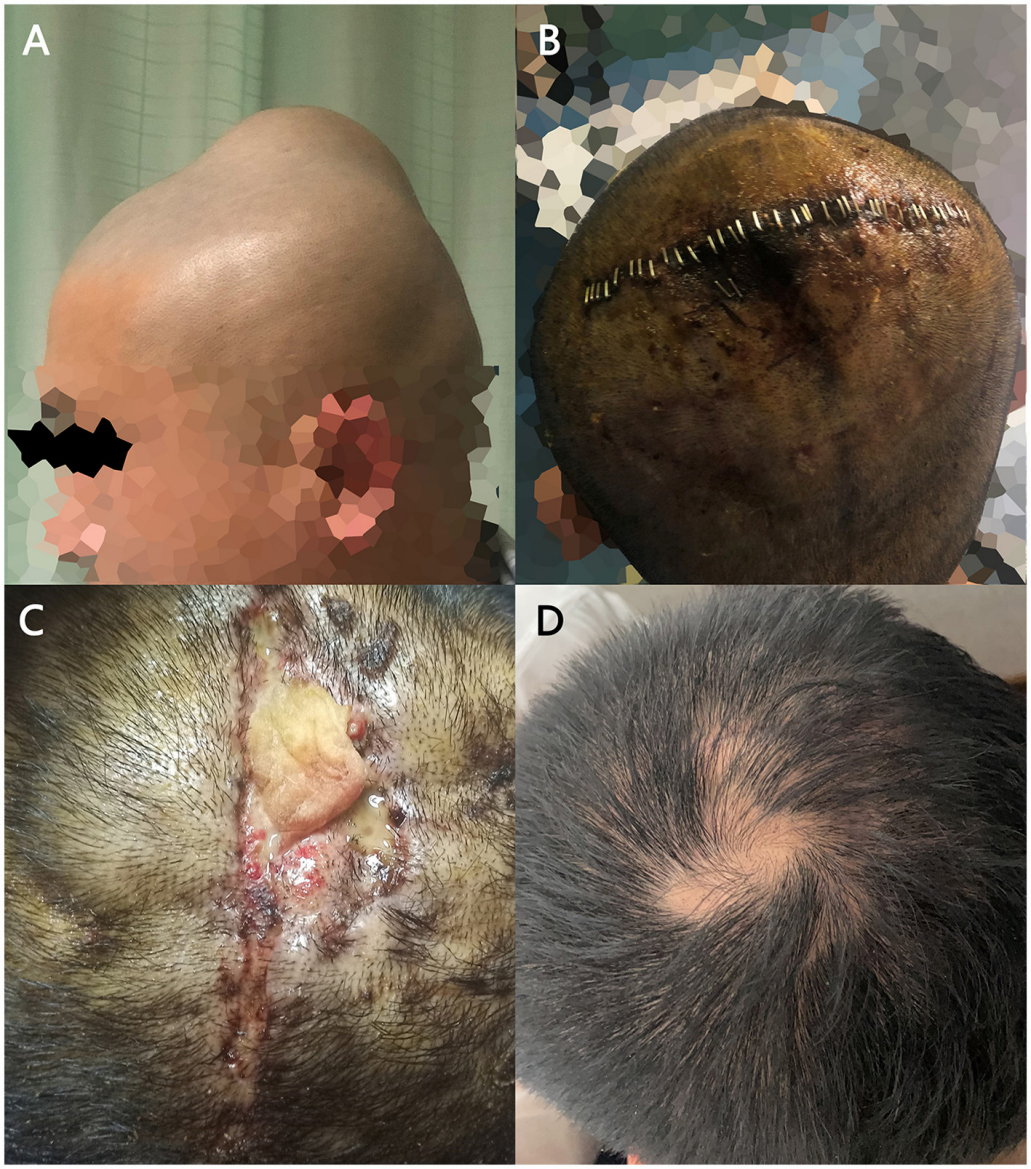
The tumor under the patient's scalp and its treatment process. **(A)** The subcutaneous mass on the top of patient's head. **(B)** The incision after the procedure. **(C)** Ruptured incision after removal of stitches with a yellow lump detectable inside. **(D)** The patient's healing incision in the follow-up visit.

A CT scan of the skull was performed, revealing a cutaneous mass above the parietal bone with a potential lytic abnormality of the bone window. MRI showed that the lesion had not infiltrated the dura or penetrated the central nervous system (CNS). The images are shown in Figure 1. We conducted relevant examinations on important parts, including CT scans of the head and chest, MR scans of the head, and Color Ultrasound of the abdomen. No obvious abnormalities were found. Besides, we conducted a physical examination of the patient, and no abnormal skin and lymph node changes were detected in the patient's trunk and limbs.

**Figure 2:**
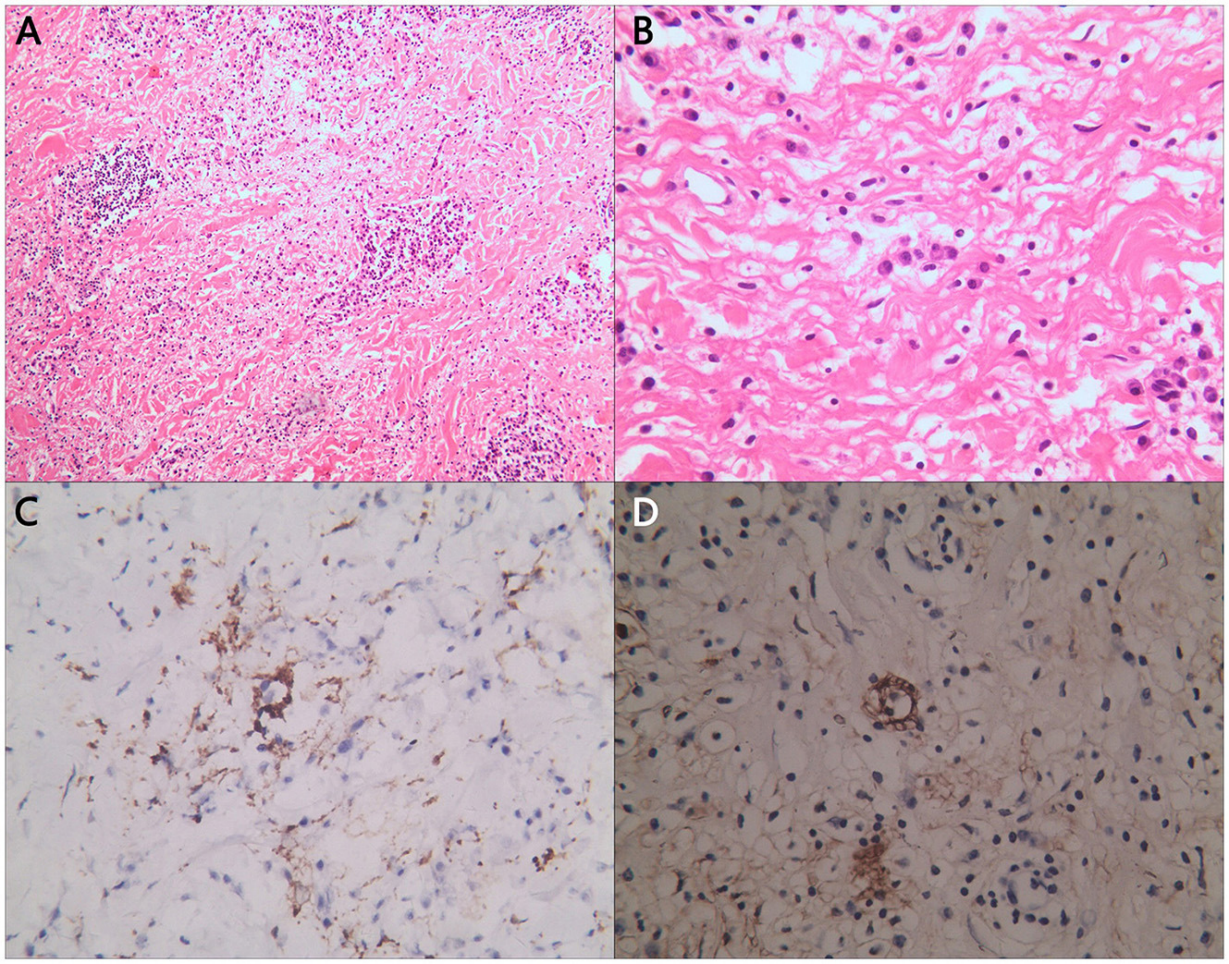
Partial pathological section of tumor. Histological images of **(A)** H&E staining of enlarged histiocytes admixed with other inflammatory cells; **(B)** some enlarged histiocytes were observed phagocytosing plasmacyte; and **(C)** S100 and **(D)** CD163 immunostaining was positive in larger histiocytes.

We designed an 8 cm surgical incision in the parietal center of the scalp. During the procedure, we found that the lump had an abundant blood supply and poorly defined boundaries between the scalp and the lump, but there were no adhesions to the parietal bone. We decompressed the mass into blocks, and a total mass of 5 cm × 4 cm × 4 cm was resected with bleeding of approximately 750 ml. The patient suffered subcutaneous fluid 1 week after the operation. We aspirated 10 ml of blood-colored fluid by puncture. Then the surgical incision was dressed with a pressure bandage. The stitches were removed 10 days after surgery. The incision ruptured 20 days after initial procedure, and we observed xanthogranuloma in the fluid. Then the patient was referred to our plastic surgery department for resection of the granuloma and suturing of the incision. Figure 2 shows the treatment process of the patient's tumor.

**Figure 3:**
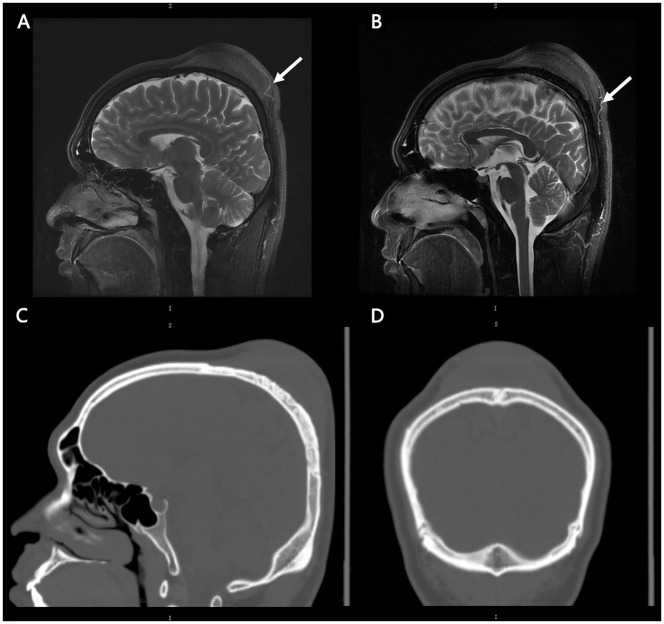
MRI and CT images of patients. Radiographic findings. Initial MRI brain sagittal cuts and T2-weighted sequences showing that the lesion did not have central nervous system involvement **(A, B)**. There was abundant blood supply from the scalp (white arrow). Initial CT brain bone window along the sagittal **(C)** and coronal **(D)** views showing the potential lytic abnormality and the low density of the lesion. No obvious calcification of the lesion was found.

The diagnosis of RDD can be made by hematoxylin-eosin (H&E) staining section and specific immunohistochemical markers. Usually, there can be observed pale histocytes and emperipolesis of plasma cells and lymphocytes. Besides, S100, CD68, CD163 and CD1a are RDD's classic markers in staining. Figure 3 demonstrates pathological sections of the patient's tumor samples. Abundant lymphocyte and plasma cell infiltration was detected in the tissue. The cytoplasm was lightly stained, the histiocytes with ovoid nuclei were scattered, and suspiciousemperipolesis. Fibrous tissue in the interstitium was significantly proliferated and collagenized.

The immunohistochemical report showed that CD68 and CD163 in tissue cells, S100 in partial tissue cells, and LCA, CD20, CD79α, CD45RO, CD3, and IgG in partial tissue cells were positive; Igλ was weakly positive; Igκ and IgG4 were negative; EMA, CD38, and CD138 in plasmacytes and VIM and CD34 in capillary walls were positive; CK, GFAP, CD1a, PAS, and antacid staining were negative, The Ki-67 staining result is 3%.

## Discussion

Originally, RDD was considered as a reactive and histioproliferative disorder of unknown cause, patients mostly being young males (male-to-female ratio: 3:1) who presenting with painless lymphadenopathy and systemic symptoms of fever and weight loss (6). The etiology of RDD remains unknow. Existing mechanisms include immune dysfunction (7) and possible viral infection such as the varicella zoster virus, herpes virus, Epstein–Barr virus or HIV (8),

However, our patient exhibited an exclusive cutaneous RDD without systemic symptoms. CRDD is easy (9) to be diagnosed pathologically, but in clinical settings, it is usually confused with lipoma and cranial osteoma (10). It is necessary to perform pathological diagnosis of the mass after excision. In this case, the patient developed an effusion under galea aponeurotica. Purulent secretions from proliferating lymphocytes increased risk of incision infection.

In previously reported cases, CRDD observed in scalp cases are rare. Eran et al. reported a 10-year-old boy presenting with multiple Langerhans cell histiocytosis of the bone and CRDD. In addition, the patient showed amelioration after prednisone and vinblastine therapy (3). Similarly, Chen et al. reported a 41-year-old man with a subcutaneous mass affecting the CNS. The patient was treated with surgical excision (4). However, the patient developed a lesion in his spine and agreed to undergo additional surgical treatment. One case similar to our patient was reported during the past few years, and doctors opted for surgical resection (11). There is still no consensus regarding the best clinical treatment for CRDD, but different methods have been reported for many types of CRDD, such as cryotherapy, radiotherapy, steroid injections, or treatment with systemic corticosteroids, retinoids, dapsone, imatinib mesylate, or thalidomide, which is recommended in the NCCN guidelines (12–15). Immunomodulatory therapy is a preferable method. Since RDD is a clonal histocyte disorder with mutation in the MAP2K1 and KRAS pathway (16), that are amenable to therapy with MEK inhibitors (17). Zhao et al. (18) reported a 54-year-old woman with reddish-brown nodules on her body, and they prefer to use lenalidomide in cutaneous disease that cannot be resected (18).

Some clinical data from cases of RDD in cutaneous tissue of the scalp with bony involvement are summarized in Table 1. The patients ranged in age from 26 to 54 years at the time of the initial occurrence. Unlike other cases reported in recent decades, our patient's lesion seemed to originate from the galea aponeurotica without calvarial or intracranial involvement, and the patient's distal lymph nodes did not show significant swelling. The patient also had no systemic symptoms. Therefore, we chose surgical excision for the lesion, but we underestimated the risk of infection and issues with incisional healing. The patient recovered after scalp debridement suturing and vacuum sealing drainage treatment. During the follow-up visit, we learnt that the lesion had not recurred in 1.5 years since the first procedure.

**Table 1:** Details of scalp CRDDs cases in recent reports.

**Patient**	**Age(years)/ Sex**	**Location**	**Presentation**	**Number of lesions**	**Size (cm)**	**Treatment**	**Follow-up**
1	51/Male	Right temporal region of the scalp	Red nodule with no symptoms	Single	2.5	None	Reduction in size
2	53/Female	Midline vertex region of the scalp	Yellow nodule with no symptoms	Multiple	1.5	Excision and declined radiation	No data
3 Griauzde et al. (19)	26/Male	Left greater wing of the sphenoid and temporal bone	Mass with no symptoms	Single	No data	Excision	No data
4 Demicco et al. (20)	45/Female	Frontoparietal bone	Yellow to white tumor with seizures, headache for 2 months	Multiple	No data	Excision	No data
5 Wenig et al. (21)	54/Female	Sphenoid bone, temporal bone, and pterygoid fossa	Decreased vision in right eye and numbness in face for 6 months	No data	No data	Excision	Death from a intracerebral hemorrhage shortly after a craniotomy during an attempt to respect the intracranial extension of disease
6 (our patient)	31/Male	Parietal region of scalp	No symptoms	Single	5.0	Excision, antibiotics, and vacuum sealing drainage	No recurrence was detected in 1.5 years

There are still limits in this study. Result from the limited objective conditions, we did not perform generation sequencing of the lesion, which might be of great help to following treatment after surgical procedure. Moreover, we report a single case, which potential provided limited treatment methods of RDD.

## Conclusions

RDD of the scalp is rare. Surgical incision can cure the lesion but it may become infected because of lymphocytic infiltration. As a consequence, early diagnosis and differential diagnosis of RDD are necessary. For treatment, individualized therapy is critical to patient prognosis.

## Data availability statement

The original contributions presented in the study are included in the article/supplementary material, further inquiries can be directed to the corresponding authors.

## Ethics statement

Written informed consent was obtained from the individual(s) for the publication of any potentially identifiable images or data included in this article.

## Author contributions

All authors listed have made a substantial, direct, and intellectual contribution to the work and approved it for publication.
